# Supplementary material for: Sequencing during Times of Change: Evaluating SARS-CoV-2 Clinical Samples during the Transition from the Delta to Omicron Wave
Source: Viruses. 2022 Jun 28;14(7):1408. doi: 10.3390/v14071408 (PMC9320617; doi:10.3390/v14071408)
Supplement: Supplementary file 1 [file viruses-14-01408-s001.zip › viruses-1744531-supplementary.pdf]

Manuscript title: Sequencing during times of change: Evaluating SARS-CoV-2 clinical samples during the transition from the Delta wave to Omicron wave in Chicago, USA

Shuchen Feng[1], Mudassir Ali[1], Monika Evdokimova[1], Gail E. Reid[2,3], Nina M. Clark[2,3], Susan L. Uprichard[1,2,3] and Susan C. Baker[1,3,*]

[1] Department of Microbiology and Immunology, Stritch School of Medicine, Loyola University Chicago, Chicago, IL 60153, USA; sfeng3@luc.edu (S.F.); mali20@luc.edu (M.S.A); mevdokimova@luc.edu (M.E.); suprichard@luc.edu (S.L.U.)
[2] Department of Medicine, Stritch School of Medicine, Loyola University Chicago, Chicago, IL 60153, USA; nmclark@lumc.edu (N.M.C.); greid@lumc.edu (G.E.R.)
[3] Infectious Disease and Immunology Research Institute, Stritch School of Medicine, Loyola University Chicago, Chicago, IL 60153, USA
* Correspondence: sbaker1@luc.edu

Content: List of Supplemental figures and tables

Figure S1. Mutation numbers identified at different reads frequencies in eight samples sequenced using both ArticV4.1 and Varskip2 primer schemes.

Figure S2 Summarized total reads depth across the genome for Omicron samples sequenced using the Varskip2 primer scheme (n=6).

Figure S3. Silent and missense mutation frequencies in groups of variants (Delta or Omicron) and patient status (ICU/not-ICU).

Table S1. Details of patient demographics (n=34), sample Ct values and lineages.

Table S2. Private mutations identified from the 37 samples by Nextclade.

Table S3. Variant-specific mutation list used for detection of potential recombination events.

Table S4. Welch's t-tset results for comparison of silent and missense mutation frequencies in available Omicron and Delta samples.

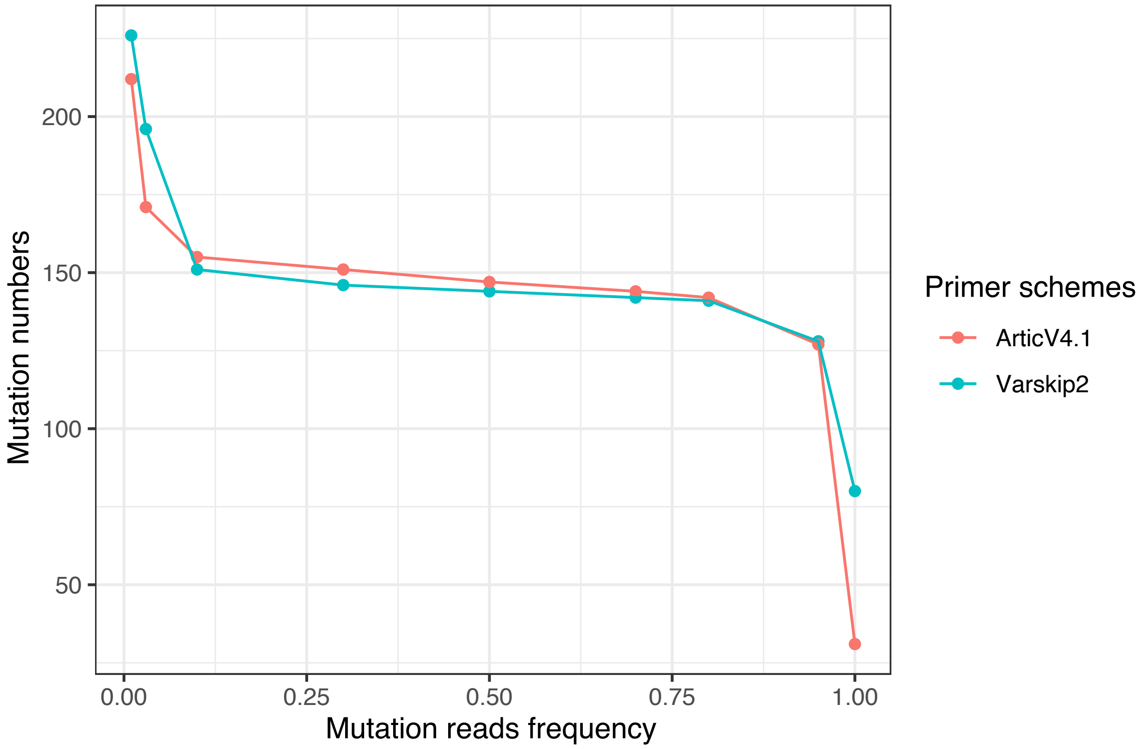

Figure S1. Mutation numbers identified at different reads frequencies in eight samples sequenced using both ArticV4.1 and Varskip2 primer schemes. Mutation numbers at reads frequency values of 0.01, 0.03, 0.1, 0.3, 0.5, 0.7, 0.8, 0.95 and 1 are plotted.

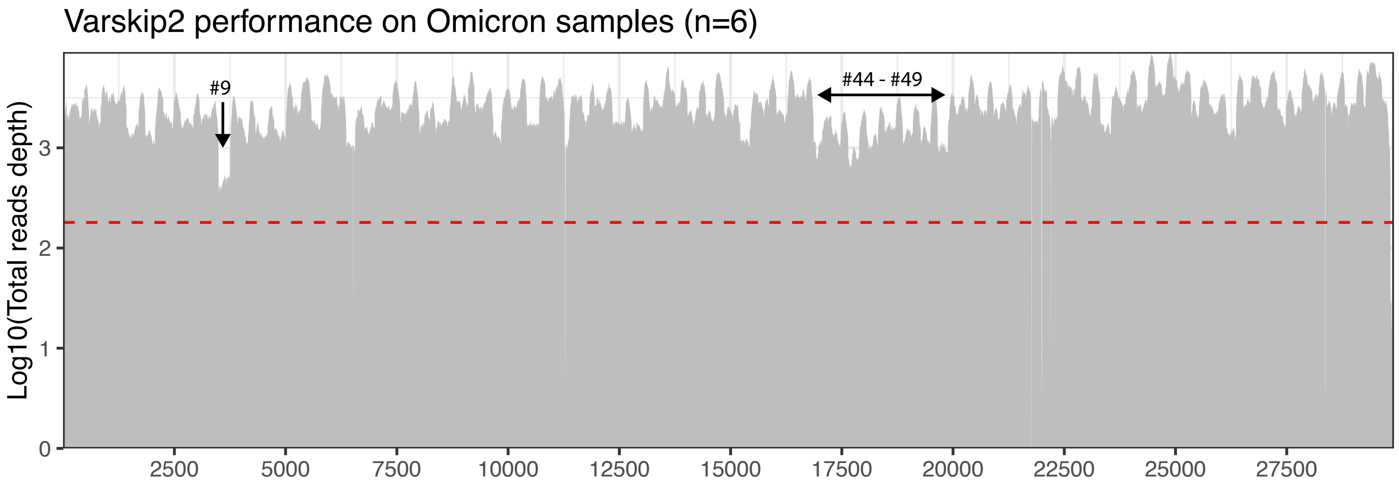

Figure S2. Summarized total reads depth across the genome for Omicron samples sequenced using the Varskip2 primer scheme (n=6). The X-axis represents for genome positions. The Y-axis represents for log10 transformed total reads depth. The relatively lower coverage amplicon regions (#9 and #44-#49) are indicated in arrows.

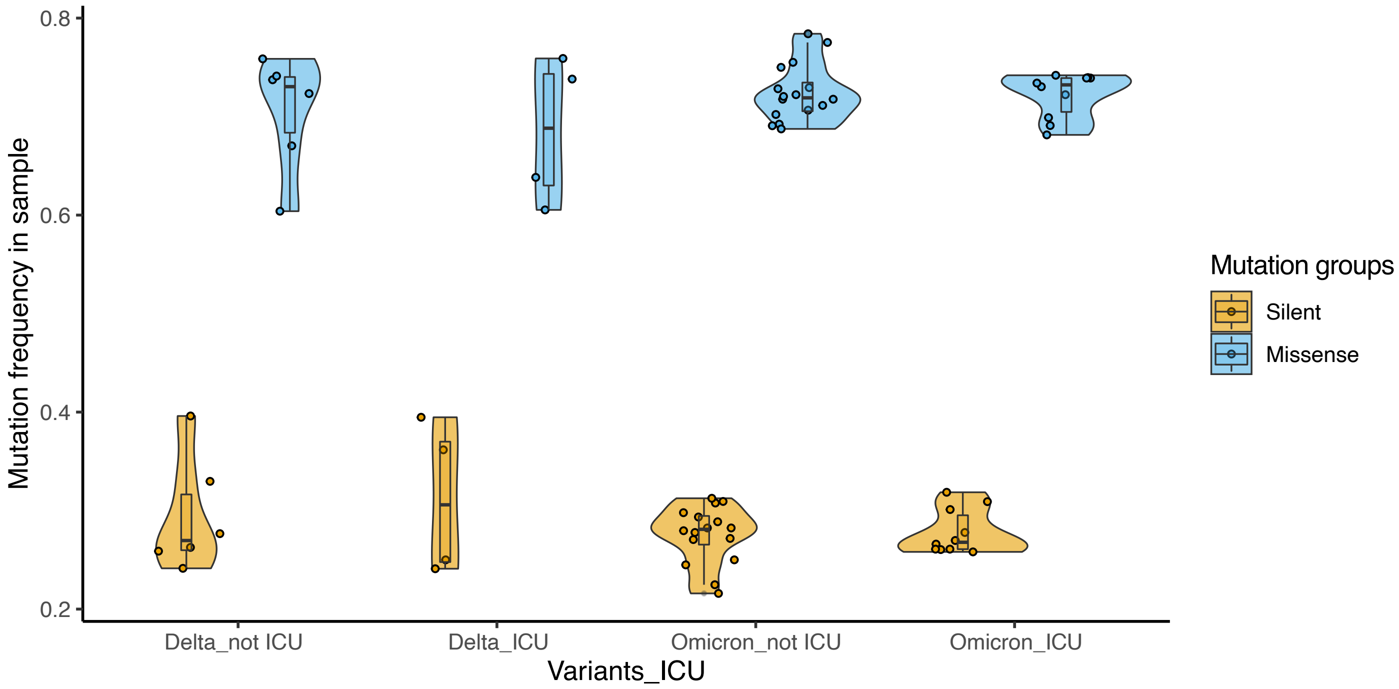

Figure S3. Silent and missense mutation frequencies in groups of variants (Delta or Omicron) and patient status (ICU/not-ICU). Silent mutations and missense mutations are represented in yellow and blue colors, respectively. The whiskers in the box plot represent for the 25th percentile, median and 75th percentile from the bottom to the top, respectively.

Table S1. Details of patient demographics (n=34), sample Ct values and lineages.

Sample ID	Sex	Age	Sampling Date	Vaccinated	Immuno-suppressed*	Hospitalized**	Viral Load (Ct)	Lineage
P1	F	62	05/26/21	Yes	Yes	Yes/ICU	21.1	Alpha(B.1.1.7)
P2	F	67	08/05/21	Yes	Yes	No	23.6	Delta(AY.110)
P3	M	52	11/03/21	Yes	No	Yes	24.5	Delta(AY.39)
P4	F	54	11/03/21	Yes	Yes	Yes/ICU	22.0	Delta(AY.25.1)
P5	M	53	11/24/21	Yes	Yes	Yes	20.4	Delta(AY.42.1)
P6	M	38	11/25/21	Yes	No	Yes	18.4	Delta(AY.3)
P7	M	52	12/07/21	Yes	Yes	Yes/ICU	26.3	Delta(AY.117)
P8	M	30	12/15/21	Yes	No	No	28.1	Omicron(BA.1)
P9	M	68	12/17/21	Yes	Yes	Yes	20.8	Delta(AY.103)
P10	M	57	12/20/21	Yes	Yes	Yes	26.4	Delta(AY.3)
P11	F	63	12/27/21	Yes	Yes	Yes/ICU	20.2	Omicron(BA.1)
P12	M	49	12/29/21	Yes	No	Yes	23.8	Omicron(BA.1.15)
P13	F	67	12/31/21	Yes	Yes	Yes	20.0	Omicron(BA.1.15)
P14-1	M	70	12/31/21	Yes	Yes	Yes/ICU	27.7	Delta(AY.103)
P14-2			01/10/22			Yes/ICU	26.1	Delta(AY.103
P15	F	30	01/01/22	Yes	Yes	Yes	33.1	Omicron(BA.1.1)
P16-1	M	78	01/04/22	Yes	Yes	No	24.5	Omicron(BA.1)
P16-2			01/18/22			Yes/ICU	26.8	Omicron(BA.1)
P17	M	66	01/05/22	Yes	Yes	Yes/ICU	22.4	Omicron(BA.1)
P18	M	58	01/05/22	Yes	No	No	30.6	Omicron(BA.1)
P19	M	59	01/06/22	Yes	Yes	No	21.5	Omicron(BA.1)
P20	F	52	01/07/22	Yes	No	No	23.6	Omicron(BA.1.1)
P21	F	61	01/09/22	Yes	Yes	No	29.7	Omicron(BA.1)
P22	F	53	01/10/22	Yes	Yes	Yes	25.4	Omicron(BA.1)
P23	F	67	01/13/22	Yes	No	Yes	29.2	Omicron(BA.1.1)
P24	F	67	01/13/22	Yes	Yes	Yes/ICU	19.0	Omicron(BA.1.1)
P25	F	64	01/13/22	Yes	Yes	Yes/ICU	27.3	Omicron(BA.1)
P26	M	62	01/14/22	No	Yes	Yes	26.9	Omicron(BA.1.15)
P27	F	65	01/14/22	Yes	Yes	Yes/ICU	22.2	Omicron(BA.1)
P28	M	71	01/15/22	Yes	Yes	Yes/ICU	19.8	Omicron(BA.1)
P29-1	F	62	01/15/22	Yes	Yes	No	27.3	Omicron(BA.1.1)
P29-2			02/01/22			Yes/ICU	28.6	Omicron(BA.1.1)
P30	M	79	01/18/22	Yes	Yes	Yes	17.8	Omicron(BA.1)
P31	M	52	01/18/22	Yes	Yes	Yes/ICU	22.0	Omicron(BA.1)
P32	M	48	01/20/22	Yes	Yes	Yes/ICU	22.6	Omicron(BA.1.1)
P33	M	41	01/24/22	Yes	Yes	Yes	24.5	Omicron(BA.1)
P34	M	40	01/31/22	Yes	Yes	Yes	25.5	Omicron(BA.1)

* Immunosuppression due to multiple myeloma, solid organ transplant, systemic lupus erythematosus, or chronic lymphoid leukemia.

** P18, P19 and P21 were asymptomatic infections admitted for non-COVID reasons, therefore were considered as not hospitalized for COVID in this study.

Table S2. Private mutations identified from the 37 samples by Nextclade. Nucleic acid changes in the first 50 bp and last 100 bp length of the reference genome are not included as these are affected a lot by low coverage and high rate of sequencing and/ or mapping errors.

Patients	Variants	Lineages	Labeled private mutations	Unlabeled private mutations	Numbers of labeled private mutations	Numbers of unlabeled private mutations
P1	Alpha	B.1.1.7	C2395T,C7851T,T15096C	C4206T,C7299T,C10138T,C11750T,C19813T	3	5
P2	Delta	AY.110		C13326T,C19154T,A27397G,G27415A,T27504A,C28744T	0	6
P3	Delta	AY.39		C2509T,C3619T,C12084T,A16006G,C22444T,T23725C,G25855C,C26111T	0	8
P4	Delta	AY.25.1		C7200T,T8764C,C13620T,G29425A	0	4
P5	Delta	AY.3		C3695T,C4012T,C7162T,G7246T,A11782G,C12073T,C12513T,T13701C,C15141T,C24374T,G27390T,G27589T,C28313T	0	13
P6	Delta	AY.3		C1479T,C9073T,G10533A,A10994G,C18912T,G18948T,C21145T,C22480T,C26936T	0	9
P7	Delta	AY.117		C3315T,G10642A,C22432T,C27304T	0	4
P8	Omicron	BA.1	C28472T	C10543T,C11950T,A21137G,T23332C,C29144T	1	5
P9	Delta	AY.103	C18744T	A259G,C4900T,G6671A,C14688T,C14925T,G19684T,C21910T,G23401T,G26828A,G27260T,C28236T	1	11
P10	Delta	AY.3		A1622T,C5565T,T5566A,C5730T,C6681T,G21055A,C22624T,G24390T,A27663G,G29734T,G29742T	0	11
P11	Omicron	BA.1		A8590G	0	1
P12	Omicron	BA.1.15		C25777T	0	1
P14-1	Delta	AY.103	C9891T	C664T,C2842T,T6661C,C12890T,T14448C,A22780T,G22899T,A23013T,C24034T,G27967A,G29422A	1	11
P14-2	Delta	AY.103	C9891T	C664T,C2842T,T6661C,C12890T,T14448C,A22780T,G22899T,A23013T,C24034T,G27967A,G29422A	1	10
P15	Omicron	BA.1.1	C21595T		1	0
P16-1	Omicron	BA.1	C28472T	C6361T,C11950T	1	2
P16-2	Omicron	BA.1	C28472T	C6361T,C11950T	1	2
P17	Omicron	BA.1	C28472T	T6152C,A6293G,A7930G,G8371A,G11534A,C11950T	1	6
P18	Omicron	BA.1	C28472T	C11950T,C17898T,C25515T	1	3
P19	Omicron	BA.1		C25714T	0	1
P20	Omicron	BA.1.1	C21595T	C18788T	1	1
P21	Omicron	BA.1	C28472T	A7981G,C11950T,T22685A,T22686A	1	4
P22	Omicron	BA.1	C28472T	C11950T	1	1
P24	Omicron	BA.1.1	C21595T		1	0
P25	Omicron	BA.1	C28472T	C11950T,C22224T	1	2
P26	Omicron	BA.1.15		G14268A,C19572T	0	2
P27	Omicron	BA.1	C28472T,A28877T,G28878C	C4406T,C11950T,C26447T	3	3
P28	Omicron	BA.1	C28472T	C11950T	1	1
P29-1	Omicron	BA.1.1	C21595T	C676T,A22005C,C24334A	1	3
P29-2	Omicron	BA.1.1	C4965T,C21595T,G23012A	T168C,C676T,G4985A,C12784T,A22005C,G23282A,C24334A	3	7
P30	Omicron	BA.1	C28472T	A6625T,C9207A,C11950T,C17135T	1	4
P31	Omicron	BA.1	C28472T	G3122T,C11950T,T28642C	1	3
P32	Omicron	BA.1.1	C21595T	C15928T,T27569C	1	2
P33	Omicron	BA.1	C28472T	C3923T,C11950T	1	2
P34	Omicron	BA.1	C28472T	C11950T,C17402A,G19851A	1	3

Table S3. Variant-specific mutation list for detection of potential recombination events [1-3].

Genomic position	Nucleic acid change	gene	Index for lineage	Used for Delta v.s. Omicron	Used for BA.1 v.s. BA.2
670	T670G	ORF1ab	BA.2, BA.3	yes	yes
2790	C2790T	ORF1ab	BA.2	yes	yes
2832	A2832G	ORF1ab	BA.1, BA.1.1	yes	yes
4184	G4184A	ORF1ab	BA.2, BA.3	yes	yes
6402	C6402T	ORF1ab	Delta	yes	
7124	C7124T	ORF1ab	Delta	yes	
8393	G8393A	ORF1ab	BA.1, BA.1.1	yes	yes
9053	G9053T	ORF1ab	Delta	yes	
9344	C9344T	ORF1ab	BA.2	yes	yes
9534	C9534T	ORF1ab	BA.2, BA.3	yes	yes
9866	C9866T	ORF1ab	BA.2	yes	yes
10449	C10449A	ORF1ab	BA.1, BA.1.1, BA.2, BA.3	yes	
11201	A11201G	ORF1ab	Delta	yes	
11235	C11235T	ORF1ab	BA.3	yes	
11287	G11287T	ORF1ab	BA.1, BA.1.1, BA.2	yes	
11288	del11288_11296	ORF1ab	BA.1, BA.1.1, BA.2, BA.3	yes	
11537	A11537G	ORF1ab	BA.1, BA.1.1	yes	yes
15451	G15451A	ORF1ab	Delta	yes	
16466	C16466T	ORF1ab	Delta	yes	
17410	C17410T	ORF1ab	BA.2	yes	yes
18163	A18163G	ORF1ab	BA.1, BA.1.1, BA.2, BA.3	yes	
19220	C19220T	ORF1ab	Delta	yes	
19955	C19955T	ORF1ab	BA.2	yes	yes
21618	C21618T	S	BA.2	yes	yes
21618	C21618G	S	Delta	yes	
22578	G22578A	S	BA.1, BA.1.1, BA.2, BA.3	yes	
22599	G22599A	S	BA.1.1	yes	yes
22674	C22674T	S	BA.2, BA.3	yes	yes
22679	T22679C	S	BA.1, BA.1.1, BA.2, BA.3	yes	
22686	C22686T	S	BA.1, BA.1.1, BA.2, BA.3	yes	
22688	A22688G	S	BA.2	yes	yes
22775	G22775A	S	BA.2, BA.3	yes	yes
22786	A22786C	S	BA.2	yes	yes
22882	T22882G	S	BA.1, BA.1.1, BA.2, BA.3	yes	
22898	G22898A	S	BA.1, BA.1.1, BA.3	yes	
22917	T22917G	S	Delta	yes	
23013	A23013C	S	BA.1, BA.1.1, BA.2, BA.3	yes	
23040	A23040G	S	BA.1, BA.1.1, BA.2, BA.3	yes	
23048	G23048A	S	BA.1, BA.1.1	yes	yes
23055	A23055G	S	BA.1, BA.1.1, BA.2, BA.3	yes	
23063	A23063T	S	BA.1, BA.1.1, BA.2, BA.3	yes	
23075	T23075C	S	BA.1, BA.1.1, BA.2, BA.3	yes	
23525	C23525T	S	BA.1, BA.1.1, BA.2, BA.3	yes	
23599	T23599G	S	BA.1, BA.1.1, BA.2, BA.3	yes	
23604	C23604A	S	BA.1, BA.1.1, BA.2, BA.3	yes	
23604	C23604G	S	Delta	yes	
23854	C23854A	S	BA.1, BA.1.1, BA.2, BA.3	yes	
23948	G23948T	S	BA.1, BA.1.1, BA.2, BA.3	yes	
24410	G24410A	S	Delta	yes	
24424	A24424T	S	BA.1, BA.1.1, BA.2, BA.3	yes	
24469	T24469A	S	BA.1, BA.1.1, BA.2, BA.3	yes	
24503	C24503T	S	BA.1, BA.1.1	yes	yes
25469	C25469T	ORF3a	Delta	yes	
26270	C26270T	E	BA.1, BA.1.1, BA.2, BA.3	yes	
26530	A26530G	M	BA.1, BA.1.1	yes	yes
26577	C26577G	M	BA.1, BA.1.1, BA.2, BA.3	yes	
26709	G26709A	M	BA.1, BA.1.1, BA.2, BA.3	yes	
26767	T26767C	M	Delta	yes	
27638	T27638C	ORF7ab	Delta	yes	
27752	C27752T	ORF7ab	Delta	yes	
28311	C28311T	N	BA.1, BA.1.1, BA.2, BA.3	yes	
28362	del28362_28370	N	BA.1, BA.1.1, BA.2, BA.3	yes	
28461	A28461G	N	Delta	yes	
28881	G28881A	N	BA.1, BA.1.1, BA.2, BA.3	yes	
28881	G28881T	N	Delta	yes	
28882	G28882A	N	BA.1, BA.1.1, BA.2, BA.3	yes	
28883	G28883C	N	BA.1, BA.1.1, BA.2, BA.3	yes	
29510	A29510C	N	BA.2, BA.3	yes	yes
22673, 22674	T22673C, C22674T	S	BA.1, BA.1.1	yes	yes

Table S4. Welch's t-tset results for comparison of silent and missense mutation frequencies in available Omicron and Delta samples.

Mutation type	Group 1	Group 2	Group 1 sample numbers	Group 2 sample numbers	statistic	df	p-value	significance
Silent	Delta_not_ICU	Delta_ICU	6	4	-0.3869703	5.219775	0.714	not significant
Silent	Delta_not_ICU	Omicron_not_ICU	6	16	0.7485096	5.939187	0.483	not significant
Silent	Delta_not_ICU	Omicron_ICU	6	10	0.6418841	5.927932	0.545	not significant
Silent	Delta_ICU	Omicron_not_ICU	4	16	0.9155498	3.207296	0.423	not significant
Silent	Delta_ICU	Omicron_ICU	4	10	0.848647	3.206737	0.455	not significant
Silent	Omicron_not_ICU	Omicron_ICU	16	10	-0.260579	22.501685	0.797	not significant
Missense	Delta_not_ICU	Delta_ICU	6	4	0.4650685	5.382407	0.66	not significant
Missense	Delta_not_ICU	Omicron_not_ICU	6	16	-0.7485096	5.939187	0.483	not significant
Missense	Delta_not_ICU	Omicron_ICU	6	10	-0.6418841	5.927932	0.545	not significant
Missense	Delta_ICU	Omicron_not_ICU	4	16	-1.0287225	3.224352	0.375	not significant
Missense	Delta_ICU	Omicron_ICU	4	10	-0.959252	3.223701	0.404	not significant
Missense	Omicron_not_ICU	Omicron_ICU	16	10	0.260579	22.501685	0.797	not significant

Reference

1. Bolze, A.; White, S.; Basler, T.; Rossi, A.D.; Roychoudhury, P.; Greninger, A.L.; Hayashibara, K.; Wyman, D.; Kil, E.; Dai, H.; et al. Evidence for SARS-CoV-2 Delta and Omicron Co-Infections and Recombination. medRxiv 2022, 2022.03.09.22272113.
2. Abbas, Q.; Kusakin, A.; Sharrouf, K.; Jyakhwo, S.; Komissarov, A.S. Follow-up Investigation and Detailed Mutational Characterization of the SARS-CoV-2 Omicron Variant Lineages (BA.1, BA.2, BA.3 and BA.1.1). bioRxiv 2022, 2022.02.25.481941, doi:10.1101/2022.02.25.481941.
3. Proposal to split B.1.1.529 to incorporate a newly characterised sibling lineage. Available online: https://github.com/cov-lineages/pango-designation/issues/361 (accessed on 20 April 2022)
